# Microstructural Effects of Sulphate Attack in Sustainable Grouts for Micropiles

**DOI:** 10.3390/ma9110905

**Published:** 2016-11-08

**Authors:** José Marcos Ortega Álvarez, María Dolores Esteban Pérez, Raúl Rubén Rodríguez Escribano, José Luís Pastor Navarro, Isidro Sánchez Martín

**Affiliations:** 1 Departamento de Ingeniería Civil, Universidad de Alicante, Ap. Correos 99, 03080 Alacant/Alicante, Spain; joseluis.pastor@ua.es (J.L.P.N.); isidro.sanchez@ua.es (I.S.M.); 2 Departamento de Ingeniería Civil, Urbanismo y Aeroespacial, Escuela de Arquitectura, Ingeniería y Diseño, Universidad Europea, c/Tajo s/n, 28670 Villaviciosa de Odón, Spain; mariadolores.esteban@universidadeuropea.es (M.D.E.P.); raulruben.rodriguez@universidadeuropea.es (R.R.R.E.)

**Keywords:** micropiles, special geotechnical works, impedance spectroscopy, microstructure, fly ash, ground granulated blast furnace slag, sulphate attack, cement grouts

## Abstract

Nowadays, the use of micropiles has undergone a great development. In general, they are made with cement grout, reinforced with steel tubing. In Spain, these grouts are prepared using OPC, although the standards do not forbid the use of other cements, like sustainable ones. Micropiles are in contact with soils and groundwater, in which the presence of sulphates is common. Their deleterious effects firstly affect to the microstructure. Then, the aim of this research is to study the effects of sulphate attack in the microstructure of micropiles grouts, prepared with OPC, fly ash and slag commercial cements, compared to their behaviour when they are exposed to an optimum hardening condition. The microstructure evolution has been studied with the non-destructive impedance spectroscopy technique, which has never been used for detecting the effects of sulphate attack when slag and fly ash cements are used. Its results have been contrasted with mercury intrusion porosimetry and “Wenner” resistivity ones. The 28-day compressive strength of grouts has been also determined. The results of microstructure characterization techniques are in agreement, although impedance spectroscopy is the most sensitive for following the changes in the porous network of grouts. The results showed that micropiles made using fly ash and slag cements could have a good performance in contact with aggressive sodium sulphate media, even better than OPC ones.

## 1. Introduction

In recent years, special geotechnical works have undergone a great development, becoming essential tools thatallow solving engineering problems, mainly related to interaction between the structures and terrain. One of the most used special geotechnical works are micropiles. They have many advantages, such as adaptability to all kind of terrains [1,2]. For this reason, micropiles are used for underpinning existing foundations, supporting the crown of tunnels and stabilizing slopes and excavations, among other applications [1,2]. Micropiles are small-diameter piles with diameters under 300 mm, drilled and grouted with cement grout or mortar, reinforced with steel tubing and sometimes strengthened with one or several ribbed bars [1]. The micropiles standards [3,4] do not specify the cement type to use, provided that it reaches a certain compressive strength.

However, at least in Spain, generally the cement grouts for micropiles are usually prepared using an ordinary Portland cement, despite no existing specific restriction for preparing these grouts with other types of cements, like sustainable ones. These sustainable cements in most cases contain active additions, like fly ash and ground granulated blast furnace slag. The use of these cements shows many advantages [5,6,7,8,9]. On the one hand, these cements contribute to reduce CO_2_ emissions produced by the cement industry, because the additions replace a proportion of clinker, so less quantity of this product is needed. Furthermore, the most used active additions are wastes of other industrial processes, and their reuse in cement manufacture constitutes in itself an environmental benefit. On the other hand, the most common active additions improve the properties of cement-based materials and their effects are still now a topic of study [5,10,11,12]. Among the different active additions, in this research cements that incorporate fly ash and ground granulated blast furnace slag have been studied. Regarding blast furnace slag, the development of their hydration reactions entails the formation of additional CSH phases, increasing the microstructure refinement [13], which improves the service properties of cement-based materials [14,15]. On the other hand, fly ash has a similar effect in the microstructure and properties of these materials, although this admixture is able to react with portlandite [11], which is formed during the clinker hydration. As products of these pozzolanic reactions, new hydrated phases are produced, which densify the pore network of the material [5,11]. Therefore, both additions have good performance for many applications [5], particularly for marine structures [7,16,17,18]. Despite that, cements containing fly ash or slag are not used for making cement grouts for micropiles.

With respect to the durability of micropiles, it is important to emphasize the abovementioned fact that the reinforcement elements of this type of special foundation are embedded in cement grouts (pastes), instead of concrete, as happens in the majority of civil engineering structures. Then, it could entail a different behaviour of these elements against the attack of aggressive substances, especially if sustainable cements are used.

One of the most important aggressive attacks to which micropiles can be exposed is that produced by sulphate, because this aggressive is commonly present in soils and groundwater. The sulphate attack is produced by a complex mechanism, which involves different chemical reactions between components of cement paste microstructure and sulphate ions [19]. First of all, the effect of sulphate attack produces a gradual dissolution of portlandite and decomposition of the CSH phases [19,20] and whenever the attack is being developing, more and more ettringite crystals are formed in the pore network [21]. These crystals are expansive, and when the pores are filled enough, they start to cause volumetric strains in the hardened material, which brings microcracking and a loss of pore refinement [20,22]. Moreover, as consequence of those damages in the microstructure, it is also produced a loss of strength and durability in the cement-based materials exposed to sulphate attack [22]. In relation to mineral admixtures, several studies have shown that they reduce the degree of damage produced by sulphate attack [19,23,24,25]. In general, this better behaviour has been related to the higher microstructure refinement, less C3A availability and additional formation of CSH phases [19,25] produced by the addition of fly ash and slag. Besides, for fly ash cements, the lower presence of portlandite due to their consumption during pozzolanic reactions [19,26] also favours their better sulphate resistance. Nevertheless, it has been observed that the performance against sulphate attack of sustainable cements depends on the kind of active addition used, and the degree of substitution of clinker by admixture [25,27,28,29,30,31,32].

As has been previously mentioned, the microstructure of cement-based materials is directly related to their service properties [13,33], especially their durability. Nowadays, the use of non-destructive techniques for characterizing the microstructure of cement-based materials has become an important research field [34,35,36,37,38,39]. Among these techniques, impedance spectroscopy has experienced a great development during the last years. This technique is based on the possibility of correlating dielectric and mechanical properties of a solid material [40]. Recently, impedance spectroscopy has been successfully used for studying the microstructure of cement-based materials [41,42,43,44,45], although there is no experience of using this technique for detecting the effects of sulphate attack when cements which incorporate fly ash and slag are used. However, it has been demonstrated that impedance spectroscopy can be useful for detecting the appearance of microcracking [45]. One of the effects of the expansive forces produced by the formation of ettringite crystals is microcracking, as has been already explained, so it seems that this technique could be useful in the context of this research.

Therefore, the main objective of this research is to study the effects of sulphate attack in the microstructure of sustainable grouts for micropiles, prepared with fly ash and slag commercial cements, compared to their behaviour when they are exposed to an optimum hardening condition. Moreover, for both conditions OPC grouts have also been studied. The evolution of the microstructure has been studied using non-destructive impedance spectroscopy technique. In order to check the validity of their results, they have been contrasted with those obtained using the classical destructive technique of mercury intrusion porosimetry and the well-known non-destructive “Wenner” resistivity test. Finally, 28-day compressive strength of grouts has also been determined, because it is the main parameter established by micropiles standards [3,46,47] for validating the grouts, and in addition to this, it is a commonly used parameter in literature for analysing the performance of cement-based materials against sulphate attack [19,48,49,50].

## 2. Materials and Methods

### 2.1. Sample Preparation

Cement grouts (pastes) were studied in this research. Three different commercial cements were used for preparing the grouts. The first one was an ordinary Portland cement, designated CEM I 42.5 R (CEM I hereafter) according to the Spanish and European standard UNE-EN 197-1 [4]. Moreover, the grouts were also made with two cements which contribute to sustainability, a type III/B 42.5 L/SR [4] (CEM III from now on), with a content of ground granulated blast-furnace slag between 66% and 80% of total binder), and a fly ash cement, type CEM IV/B(V) 32.5 N [4] (labelled CEM IV hereafter), whose content of addition was between 36% and 55% of total binder. The use of commercial cements is focused on approaching to in situ conditions of micropiles construction, where it would be very difficult to prepare mixes of ordinary Portland cement and the corresponding active addition for grouting these elements. For all the grouts, the selected water to cement ratio was 0.5, which achieves the requirements of micropiles standard [3] related to this parameter, according to the results obtained in previous researches of the authors [51,52,53], in which similar grouts were characterized in depth. The mix proportions of the grouts per cubic meter are shown in Table 1.

Different types of specimens were prepared. On the one hand, three kinds of cylindrical specimens were made. They were cast in moulds of 10 cm diameter and 15 cm height and in moulds of 30 cm height and 7.5 cm or 15 cm diameter, respectively. On the other hand, prismatic specimens with dimensions 4 cm × 4 cm × 16 cm were also made [54]. All the samples were cured for 7 days in a temperature and humidity controlled chamber at 20 °C and 95% RH, and, following this period, de-moulded and introduced into the corresponding exposure medium. Before being exposed to the hardening media, 15 cm-height cylindrical samples were cut to obtain two kinds of disks of approximately 1 and 2 cm thickness. The different samples used for each technique and exposure media are summarized in Table 2.

### 2.2. Exposure Media

When the curing period had finished, all specimens were exposed to two different media. The first one was a solution which contained a 15% by weight of reagent grade anhydrous sodium sulphate (Na_2_SO_4_) in order to study the behaviour of grouts regarding the attack of this aggressive. The second medium consisted in submerging the samples in distilled water until the testing age. This last condition has been used as a reference for comparing the effects of sulphate attack to an optimum hardening condition.

With respect to the type of specimens put in contact with each medium, the 1 cm-thickness and 10 cm-diameter disks and the 30 cm-height and 15 cm-diameter cylinders were kept in distilled water, while the 2 cm-thickness disks and 7.5 cm-diameter cylinders were exposed to Na_2_SO_4_ solution. Finally, prismatic samples were used for both conditions.

For specimens immersed in distilled water, the tests were performed at different ages until 90 days, and in the case of specimens submerged in sodium sulphate solution, the maximum testing age was 120 days. This is due to the fact that it is considered that after 90 hardening days exposed to an optimum laboratory condition, like distilled water, the majority of the clinker hydration has been developed [11,55], so the microstructure would be mature enough. Furthermore, with respect to sustainable cements, 90 days is also an adequate target age for evaluating the pozzolanic activity of active additions [11]. On the other hand, in order to observe more effects of sulphate attack on grouts, which could be still relatively low at 90 days of exposure [56,57,58], despite the moderately high content of aggressive in the medium, the study time for this medium was extended until 120 days. The sodium sulphate solution was changed once during the test period, after 60 days exposure.

Finally, for both conditions, the volume of distilled water or sulphate solution was approximately 4 times the volume of the samples, as recommend the ASTM C 1012-04 standard [59].

### 2.3. Impedance Spectroscopy

Impedance spectroscopy is a non-destructive technique, which permits to follow the evolution of the microstructure of the same sample during the study period. Moreover, this technique allows obtaining global information of the microstructure of the sample, which is an important advantage compared to other classical techniques, like mercury intrusion porosimetry. Impedance spectroscopy has been mainly used for studying the pore structure of ordinary Portland cement-based materials [43,60], although several researches [45,61] have been recently published in which the microstructure evolution of samples with active additions has been followed with this technique. Nevertheless, there is no experience about using impedance spectroscopy for studying slag and fly ash cement samples exposed to sulphate attack.

The impedance analyser used for performing the measurements on cement grouts was an Agilent 4294A (Agilent Technologies, Kobe, Japan). This analyser allows capacitance measurements in the range from 10^–14^ F to 0.1 F, and its maximum resolution is 10^–15^ F. For all measurements, the electrodes used were circular (Ø = 8 cm) and made of flexible graphite, attached to a copper piece with the same diameter, and the frequencies for obtaining the impedance spectra ranged between 100 Hz and 100 MHz.

Two measurement methods were used. The first one was a contacting method, being the electrode in direct contact with the sample. The second method consisted in a non-contacting one, in which a polyester sheet (100 μm thick) was placed between the sample and each electrode. This method allows reducing the possible contributions of the sample-electrode interface [62] and it also allows minimizing the runaway capacitance existing due to the border effect [43]. In the non-contacting method, the impedance of the polyester sheets is subtracted from the total impedance measurement, to get only the impedance response of the specimen. Finally, the answer of the specimen is transformed to a spectrum in capacities using the Cole–Cole transformation [60], as this method gives an almost capacitive impedance spectrum.

**Figure 1:**
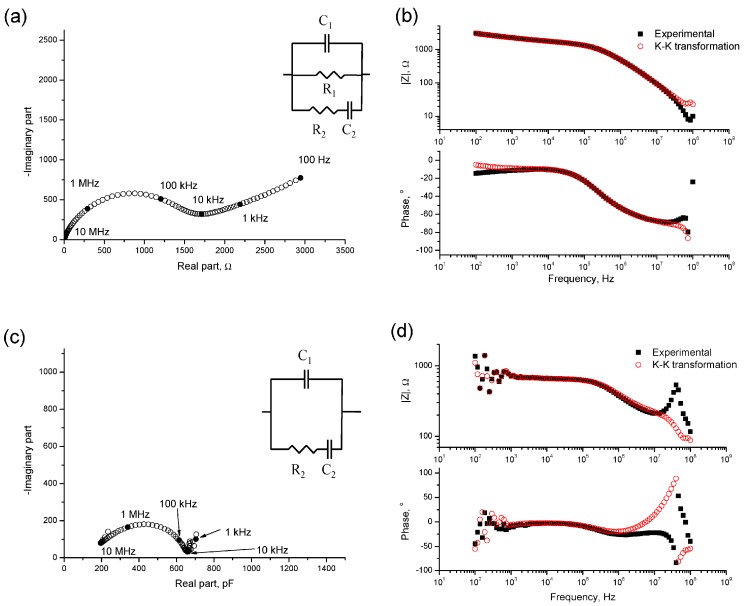
(**a**) Nyquist plot obtained for a CEM IV grout at 30 days of exposure to sodium sulphate solution and the equivalent circuit used for the fitting of the impedance spectra obtained using the contacting method [60]; (**b**) Bode plot for the same CEM IV grout validated using the Kramers–Kronig (K–K) relations (see the text for details); (**c**) Cole–Cole plot for a CEM III grout after being immersed in sodium sulphate solution during 40 days and the equivalent used for the fitting of the impedance spectra obtained using the non-contacting method [60]; and (**d**) Bode plot for the abovementioned CEM III grout validated using the Kramers–Kronig (K–K) relations.

The measurements were validated using the Kramers–Kronig (K–K) [63] (see Figure 1) and the differential impedance analysis was applied to them, in order to check the validity of equivalent circuit used [46,61,64]. The impedance spectra were fitted to the equivalent circuits proposed by Cabeza et al. [60] (see Figure 1), which included two time constants and have been used for different types of materials [43,46,61]. The fitting of the measured data to the equivalent circuits has been made using a Simplex optimization method [62]. The impedance parameters R_2_, C_1_ and C_2_ are present in both contacting and non-contacting methods. Here, only the values of these parameters obtained with non-contacting method have been studied, due to its higher accuracy.

For each cement type and exposure medium, five different samples were tested. The specimens were 10 cm-diameter disks with 1 cm of thickness for distilled water medium and with 2 cm of height in the case sodium sulphate solution. Since the thickness of the samples is not the same, the results of impedance spectroscopy cannot be directly compared. A normalization procedure already described [43,65] is necessary to establish a comparison of the evolution of the dielectric parameters as a function of the solution where samples were kept.

### 2.4. Electrical Resistivity

The electrical resistivity of the grouts was determined using the Wenner four-point test, according to the Spanish standard UNE 83988-2 [66]. This is a well-known non-destructive technique which gives information about microstructure and pore connectivity in a material [67,68]. In this research, electrical resistivity was measured on 30 cm-height with 7.5 cm- (sodium sulphate solution) and 15 cm-diameter (distilled water) cylinders using a Proceqanalyser.

### 2.5. Mercury Intrusion Porosimetry

In order to check the results of abovementioned non-destructing techniques, the microstructure of the grouts was also characterized using the classical mercury intrusion porosimetry, despite its drawbacks [69,70]. The tests were performed with a porosimeter model Autopore IV 9500 from Micromeritics (Norcross, GA, USA), which allows determining pore diameters between 5 nm and 0.9 mm. Before the test, samples were oven dried for 48 h at 50 °C. For each testing age, two measurements were made on each material, and the samples were obtained from disks of 1 cm- and 2 cm-thickness. Total porosity, pore size distribution and percentage of Hg retained at the end of the experiment were studied. For specimens immersed in distilled water the tests were performed at 2, 28 and 90 days of age, and for those exposed to sodium sulphate solution the testing ages were 28, 60, 90 and 120 days.

### 2.6. Compressive Strength

The compressive strength of the grouts is an important parameter because micropiles standard [3] establishes specific strength requirements which the grouts have to achieve. Furthermore, compressive strength is very commonly used in the literature [19,27,71] for analysing the effect of sulphate media on the behaviour of cement-based materials. The compressive strength was measured according to the Spanish standard UNE-EN 196-1 [54], using prismatic specimens 4 cm square × 16 cm deep. For each cement type and exposure medium, three samples were tested. The compressive strength was determined at 28 days of age.

## 3. Results

### 3.1. Impedance Spectroscopy

**Figure 2:**
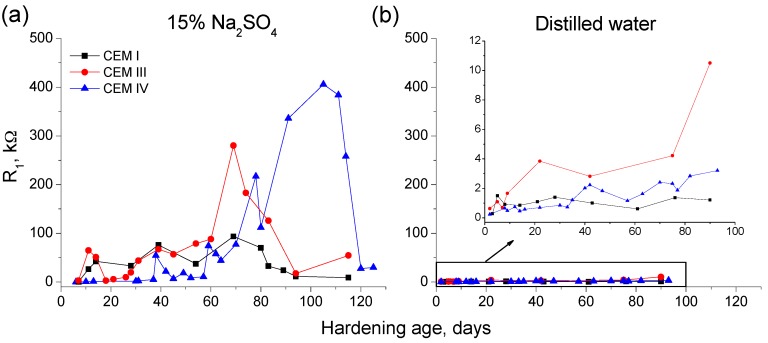
Evolution of impedance spectroscopy resistance R_1_ for CEM I, III and IV grouts exposed to: 15% Na_2_SO_4_ solution (**a**); and distilled water (**b**).

The evolution of resistance R_1_ for grouts exposed to 15% Na_2_SO_4_ solution and distilled water is depicted in Figure 2. Firstly, for samples immersed in sodium sulphate solution, it has been observed an initial increasing tendency of this parameter, although it decreased at greater ages. However, the abovementioned later fall was produced at different ages depending on the cement type used for preparing the grouts. For CEM I and III samples, the resistance R_1_ started reducing at 70 days of exposure approximately, and for CEM IV ones this resistance decreased after 110 days. In general, the R_1_ values were higher for samples with active additions. The rise of this parameter is delayed for CEM IV grouts, as consequence, until 40 days the resistance R_1_ was very similar for CEM I and III samples, with greater values than CEM IV ones. From then to 80 days approximately, CEM III grouts showed the highest R_1_ values, besides at 60 exposure days, the CEM IV grouts resistance R_1_ started rising and it overtook quickly the CEM I R_1_ values. Finally, from 80 days the greatest R_1_ were observed for CEM IV grouts, until the great fall produced at 110 days, showing similar values to CEM III samples thereafter.

In relation to the grouts submerged in distilled water during their hardening (Figure 2), the resistance R_1_ rose with time for all of them, and this parameter was higher for CEM III samples followed by CEM IV ones, so their lowest values corresponded to those prepared using CEM I. The R_1_ values were greater for grouts exposed to sodium sulphate solution than for those immersed in distilled water, which is due to the different conductivity of both media, as a consequence only the tendencies of this parameter can be compared, not their absolute values.

**Figure 3:**
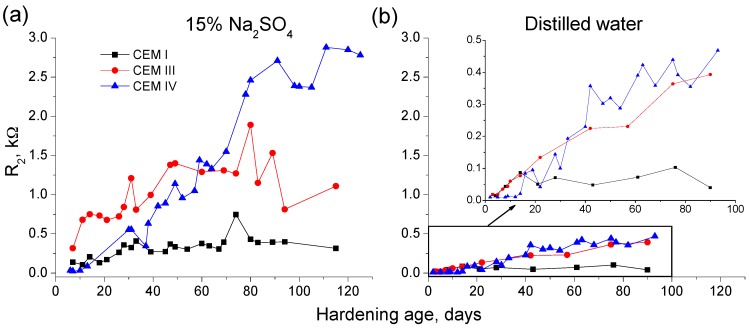
Results of resistance R_2_ for CEM I, III and IV grouts submerged in: 15% Na_2_SO_4_ solution (**a**); and distilled water (**b**).

The resistance R_2_ results for grouts hardened in contact with sulphate solution and distilled water are shown in Figure 3. In general, the tendencies observed for this parameter were very similar than those observed for resistance R_1_. The resistance R_2_ increased in the short-term for samples immersed in 15% Na_2_SO_4_ solution and it decreased at greater ages, although this late fall was less noticeable than that noted for resistance R_1_. The rise of R_2_ parameter was also slower for CEM IV grouts, as happened for R_1_. The R_2_ values were higher for grouts that incorporated active additions, in the short-term the greatest R_2_ corresponded to CEM III samples and in the long-term CEM IV ones showed the highest values of this impedance parameter.

The grouts with active additions kept in distilled water experienced an increase with time of resistance R_2_ without falling in the long-term (Figure 3), which is in accordance with R_1_ results, and, for CEM I ones, it kept practically constant. The highest R_2_ values have been observed for fly ash and slag grouts. Lastly, as it has been previously mentioned for R_1_ results, the absolute values of resistance R_2_ cannot be also compared between the both media studied, because they are influenced by their different conductivity.

**Figure 4:**
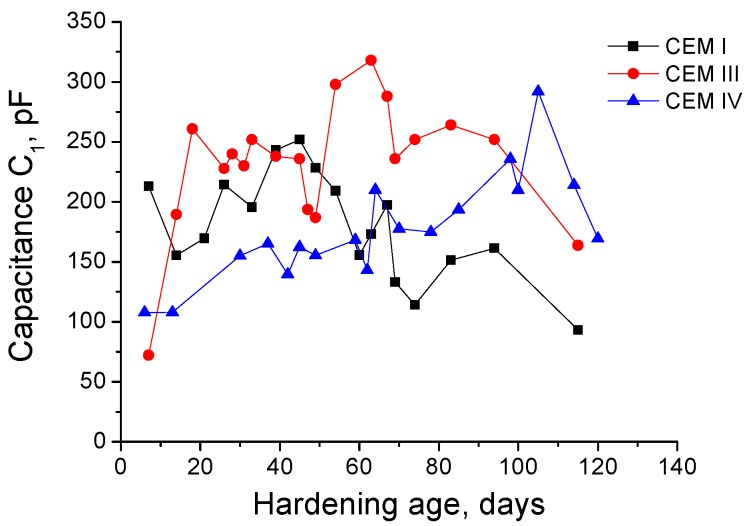
Impedance parameter C_1_ results for CEM I, CEM III and CEM IV grouts maintained in a 15% Na_2_SO_4_ solution.

The next impedance parameter results to describe are the capacitance C_1_ ones, which can be observed in Figure 4 for samples exposed to sodium sulphate solution. For all cement types studied, capacitance C_1_ showed a similar increasing tendency since early ages until it reached a maximum value, when it started decreasing. The exposure age needed for achieving this maximum depended on the cement type, for CEM I this age was 50 days approximately and it was delayed until 60 and 110 days for CEM III and IV grouts, respectively. In spite of that, in general terms, there was not a great difference between the capacitance C_1_ results obtained for the different grouts studied.

**Figure 5:**
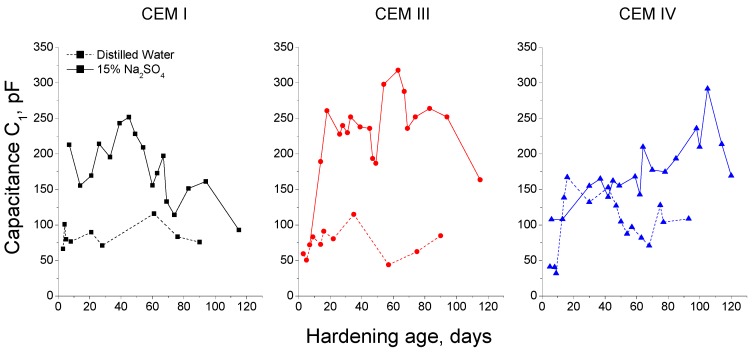
Comparison of capacitance C_1_ values obtained for CEM I, CEM III and CEM IV grouts kept in contact with distilled water (dotted line) and 15% Na_2_SO_4_ solution (continuous line).

The evolution of capacitance C_1_ of the grouts was different depending on the hardening condition, as can be observed in Figure 5. In relation to CEM I grouts, this parameter kept practically constant for those immersed in distilled water, which contrasted with the previously mentioned initial rise and the later decrease of capacitance C_1_ observed for CEM I grouts exposed to 15% Na_2_SO_4_ solution. Moreover, in general, the C_1_ values were higher for CEM I grouts in contact with sodium sulphate solution than for those kept in distilled water. The capacitance C_1_ for CEM III grouts was also greater for samples immersed in sulphate solution, however the changes with age of this parameter were more noticeable than those observed for CEM III grouts hardened in distilled water. In the short-term, the capacitance C_1_ was very similar for CEM IV samples, independently of exposure environment, and since 40 days this parameter was higher for those submerged in 15% Na_2_SO_4_ solution.

**Figure 6:**
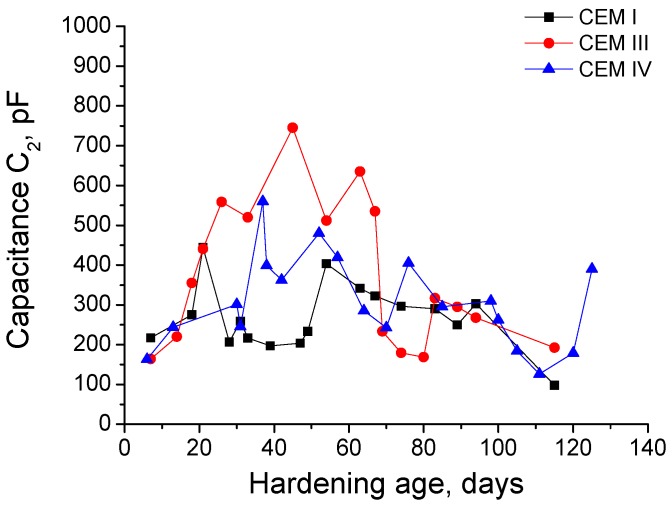
Results of capacitance C_2_ for CEM I, CEM III and CEM IV grouts exposed to 15% Na_2_SO_4_ solution.

The results of capacitance C_2_ for grouts kept immersed in sodium sulphate solution are shown in Figure 6. At early ages, this capacitance grew for the three types of grouts studied but in the long-term, it decreased for all of them, reaching similar values than the initial ones for each grout type. The highest capacitance C_2_ values corresponded to CEM III samples and the lowest were for CEM I ones.

**Figure 7:**
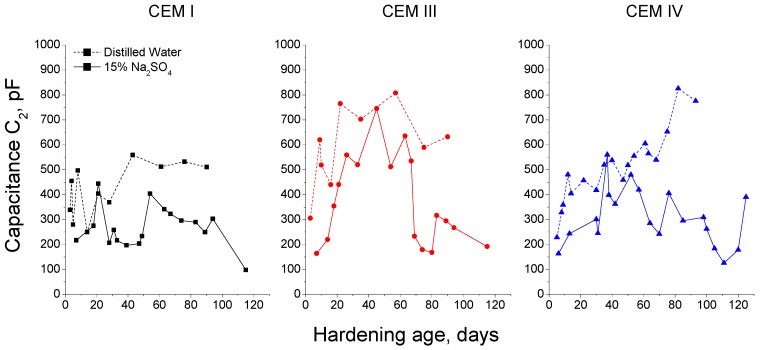
Comparison of capacitance C_2_ values obtained for CEM I, CEM III and CEM IV grouts kept in contact with distilled water (dotted line) and 15% Na_2_SO_4_ solution (continuous line).

For each cement type, the comparison between the capacitance C_2_ results obtained for grouts immersed in sodium sulphate solution and distilled water is represented in Figure 7. For CEM I samples hardened in distilled water, the capacitance C_2_ increased with time without falling at higher ages, as happened with those exposed to 15% Na_2_SO_4_ solution. The behaviour of CEM III and IV grouts regarding capacitance C_2_ was very similar than that previously described for CEM I ones, and the highest values of this parameter havebeen observed for samples immersed in distilled water.

### 3.2. Electrical Resistivity

**Figure 8:**
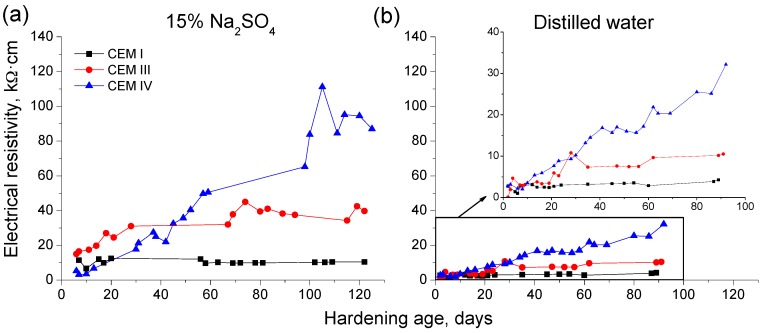
Changes with time of electrical resistivity for CEM I, III and IV grouts exposed to: 15% Na_2_SO_4_ solution (**a**); and distilled water (**b**).

The results of electrical resistivity obtained for grouts maintained in contact with sodium sulphate solution and distilled water are depicted in Figure 8. For both conditions, this parameter rose with time and it was higher for CEM IV grouts, followed by CEM III ones, and the lowest electrical resistivity values corresponded to CEM I samples. A slight decrease of this parameter has been observed for grouts which incorporated active additions exposed to sulphate solutions at later ages, although this fall was practically insignificant compared to higher decrease noted in impedance spectroscopy parameters. Finally, as happened with impedance resistances R_1_ and R_2_, the absolute values of electrical resistivity cannot be also compared between the both media studied, because they are influenced by their different conductivity.

### 3.3. Mercury Intrusion Porosimetry

**Figure 9:**
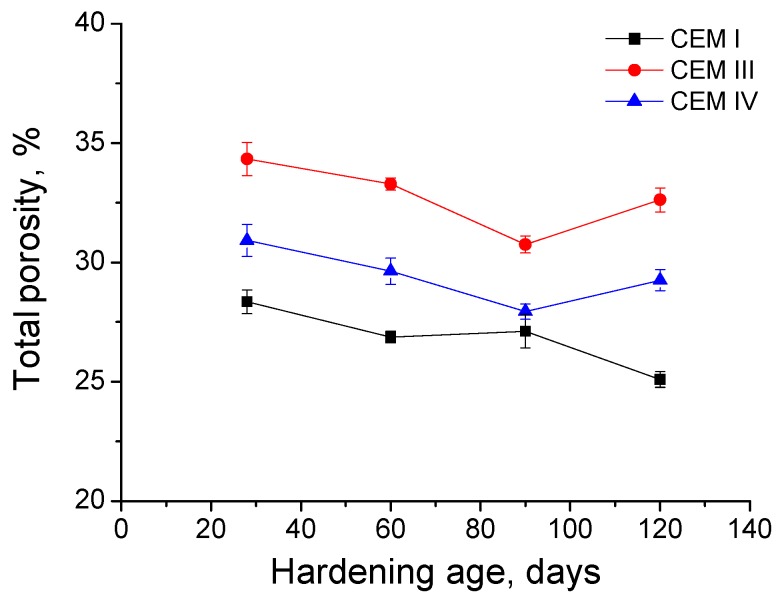
Total porosity results for CEM I, CEM III and CEM IV grouts immersed in a 15% Na_2_SO_4_ solution until 120 days.

The total porosity results for grouts exposed to sodium sulphate solution are depicted in Figure 9. In general, this parameter showed a decreasing tendency between 28 and 120 days for all cements studied. However, it has been observed a little increase of CEM I grouts total porosity between 60 and 90 days of exposure, although it returned to reduce since then until 120 days. For CEM III and IV grouts, the slight increase of this parameter was produced later, between 90 and 120 days. The lowest values of total porosity corresponded to CEM I grouts at all ages and the highest to the CEM III ones, whereas the reduction with time of this parameter was higher for grouts with slag and fly ash cements.

**Figure 10:**
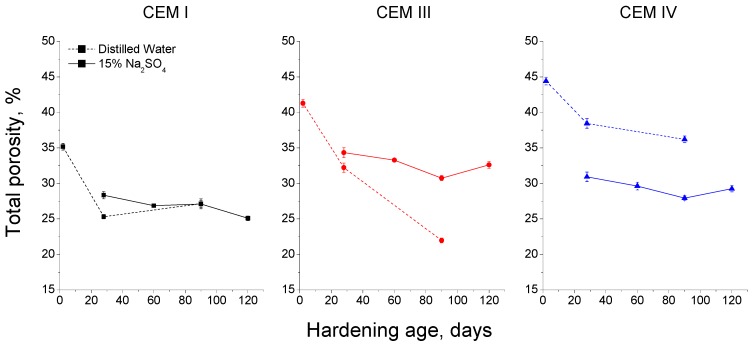
Comparison of total porosity results obtained for CEM I, CEM III and CEM IV grouts exposed to distilled water (dotted line) and 15% Na_2_SO_4_ solution (continuous line).

The total porosity comparison between grouts exposed to sodium sulphate solution and those submerged in distilled water can be observed in Figure 10. For CEM I grouts, since 28 days, the porosity is lower for those hardened in distilled water, but at 90 days of exposure there was no difference between both media. The total porosity of CEM III samples was higher for those immersed in 15% Na_2_SO_4_ solution, however, in the case of CEM IV ones, this parameter was greater for distilled water medium during all the study time.

**Figure 11:**
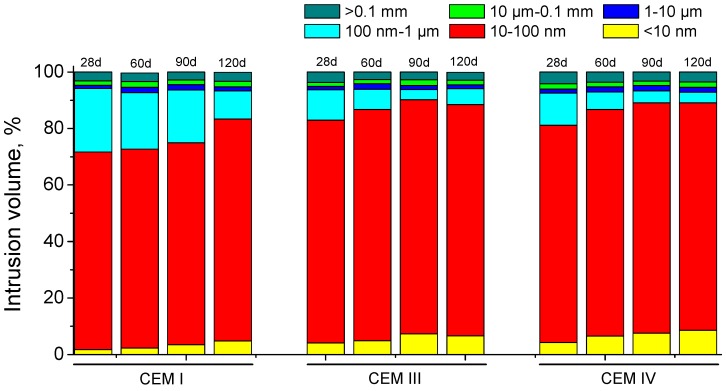
Pore size distributions (in percentage) for CEM I, III and IV grouts immersed in 15% Na_2_SO_4_ solution.

The evolution of pore size distributions for grouts exposed to sodium sulphate solution is shown in Figure 11. The pore size range between 10 and 100 nm is the majority one at all ages for the three cements studied. Furthermore, it has been observed a progressive pore refinement with time for all the grouts. The microstructure of slag and fly ash cement grouts was more refined than CEM I one, especially at early ages. For CEM III and IV grouts the main pore structure refinement was produced since 28 and 60 days, and for grouts prepared using CEM I, the major refinement has been observed between 90 and 120 days. If the pore size distributions of grouts with active additions are compared, the porous network of CEM IV grouts is a little more refined than for CEM III one, mainly at 120 days of exposure to the aggressive medium. Finally, it is important to note that CEM III grouts microstructure showed a slight loss of refinement between 90 and 120 days, as suggested the reduction of percentage of pores volume with a size less than 100 nm. For the rest of grouts, no refinement decrease has been observed in the long-term.

**Figure 12:**
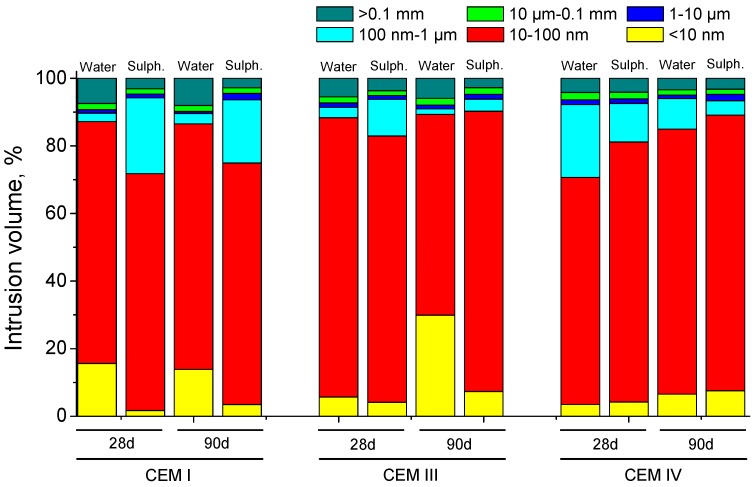
Comparison of pore sizes distributions of grouts at 28 and 90 days of immersion in distilled water and 15% Na_2_SO_4_ solution respectively.

Depending on the hardening medium, the development of grouts microstructure showed differences, as can be observed in Figure 12. For CEM I and CEM III samples, the refinement of porous network was greater for those immersed in distilled water, as stand out the relatively high percentage of pores with diameters less than 10 nm. On the other hand, for CEM IV grouts at 28 and 90 days, respectively, the microstructure was more refined for those exposed to sodium sulphate solution, showing a larger percentage of pores smaller than 100 nm.

**Figure 13:**
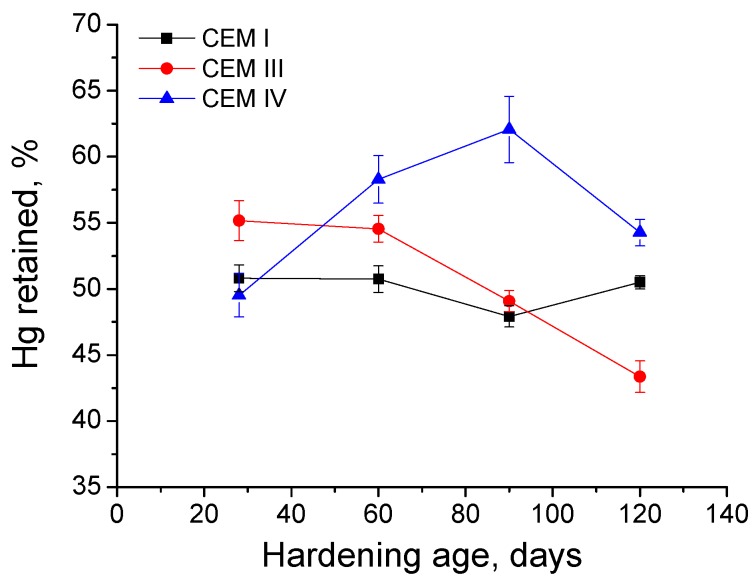
Percentage of Hg retained at the end of experiment for grouts exposed to sodium sulphate solution.

With respect to percentage of Hg retained in the samples at the end of the experiment for grouts exposed to 15% Na_2_SO_4_ solution (see Figure 13), this parameter kept practically constant for CEM I samples. Nevertheless, for CEM III ones, Hg retained decreased with time, mainly from 90 to 120 days, when it showed the lowest value of this parameter of all studied cements. The Hg retained increased until 90 days for CEM IV grouts and fell between then and 120 days, although at that age, its value was the greatest of all analysed grouts.

**Figure 14:**
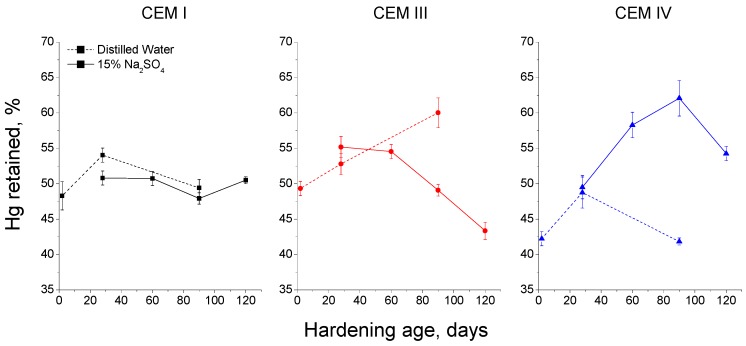
Results of percentage of Hg retained in the samples at the end of experiment obtained for CEM I, CEM III and CEM IV grouts exposed to distilled water (dotted line) and 15% Na_2_SO_4_ solution (continuous line).

If it is compared the results of Hg retained for 15% Na_2_SO_4_ solution with those obtained for distilled water (see Figure 14), several differences can be noted, especially in the case of grouts with active additions. Firstly, for CEM III grouts Hg retained increased with age for samples submerged in distilled water, which is a great difference in comparison with the important fall observed for those exposed to sodium sulphate solution. On the other hand, CEM IV grouts hardened in contact with distilled showed lower values of Hg retained than 15% Na_2_SO_4_ solution ones. Finally, for CEM I grouts there was not practically difference regarding this parameter between both exposure media studied.

### 3.4. Compressive Strength

The compressive strengths of the grouts at 28 days are compiled in Table 3. The greatest values of this parameter have been observed for samples kept in distilled water, although the difference with those exposed to 15% Na_2_SO_4_ solution was not excessive. In particular, the fall of compressive strength for samples submerged in sodium sulphate solution, compared to those hardened in water, was about 15% for CEM I grouts, 10% for CEM III ones and 4% for CEM IV ones. Moreover, the highest compressive strength corresponded to CEM I grouts and the lowest were registered for CEM IV ones.

## 4. Discussion

In relation to microstructure characterization using impedance spectroscopy, both resistances R_1_ and R_2_ are related to the electrolyte which fills the pores of the sample [60]. Particularly, the resistance R_1_ is associated with the percolating pores of the sample [60] and the resistance R_2_ is related to all the pores of the sample [60]. Therefore, it may be expected that these parameters would increase when the microstructure of the sample becomes more refined and its total porosity decreases, as a consequence of the development of hydration and pozzolanic reactions. As products of these reactions, new solid phases would be formed, which would entail a reduction of pore sizes of the samples.

The increasing tendency of resistances R_1_ (see Figure 2) and R_2_ (see Figure 3) in the short-term for the grouts exposed to sodium sulphate solution could be due to the abovementioned progressive microstructure refinement produced by the new solids formation, as consequence of the clinker and slag hydration, and the pozzolanic reactions of fly ash. The subsequent rise of both resistances for CEM IV grouts hardened in this medium, could be explained because the pozzolanic reactions of fly ash start to develop later than slag and clinker hydration [10,11], so more time is needed to observe their effects in the porous network of fly ash grouts.

The decrease of impedance resistances R_1_ and R_2_ of the grouts in the long-term (see Figure 2 and Figure 3) could indicate an increase of porosity of the samples, due the formation of expansive products as a consequence of sulphate attack. As has been previously mentioned, the resistance R_1_ is associated with percolating pores of the sample, so the aggressive ions can access directly into them, which could produce a faster and higher degree of sulphate attack. On the other hand, the resistance R_2_ includes all pores of the samples, so in addition to the accessible ones, this parameter takes into account the occluded pores, which do not received directly the sulphate attack. Then, the microstructure refinement caused by the development of hydration and pozzolanic reactions would continued in this occluded pores, entailing higher values of resistances R_2_, which would balance out the decrease of this parameter due to the destruction of microstructure produced by sulphate attack in the percolating pores. However, the lower resistance values (related to higher size pores) will be those that controlled the final resistance R_2_ results, and this could explain the fall of this parameter also noted in the long-term, although it was not as high as has been observed for resistance R_1_.

The resistances R_1_ and R_2_ showed different behaviour for grouts immersed in sodium sulphate solution than for those hardened in distilled water (see Figure 2 and Figure 3). At early ages, both resistances grew with time for grouts in contact with water, which could mean a progressive pore network refinement produced by the new solids formation as products of hydration and pozzolanic reactions. Nevertheless, it has not been observed a decrease at later ages of both impedance resistances for grouts submerged in distilled water, as happened for those exposed to sulphate attack. This result would corroborate the hypothesis that in the long-term expansive products was formed in the grouts immersed in sulphate solution, raising their porosity and reducing their microstructure refinement.

The next impedance spectroscopy results to discuss are those obtained for capacitance C_1_. This parameter is related to the solid fraction in the samples [60], so it is expected that this parameter increases as solid formation is produced due to the development of slag and clinker hydration and fly ash pozzolanic reactions. Besides, it could be also supposed that the capacitance C_1_ decreases when there is a loss of solid fraction of the samples caused by the destruction of their microstructure due to the sulphate attack.

With respect to capacitance C_1_ results for grouts in contact with sodium sulphate solution (see Figure 4), the initial increase and following decrease observed for this parameter coincides with resistance R_1_ and R_2_ results. As has been previously explained, they could be due to the early solids formation, as a consequence of hydration and pozzolanic reactions, and the later appearance of expansive products produced during the sulphate attack. Furthermore, the different age when it was reached the maximum C_1_ for each cement type, especially for CEM IV grouts, was also in accordance with R_1_ and R_2_ results, and reveals the delaying between the beginning of fly ash pozzolanic reactions and clinker and slag hydration [10,11]. The fact that there was not a great difference between the capacitance C_1_ values obtained for the different grouts and exposure conditions studied (Figure 4 and Figure 5), could indicate that the solid phase of the samples was very similar for the three cement types, independently of the different pore size distribution for each one.

Regarding the comparison between the capacitance C_1_ results for both media studied (see Figure 5), the higher values showed by CEM I grouts exposed to sulphate solution compared to distilled water ones, could be related to the silting of the pores due to the formation of ettringite. This could also explain the similar result observed for CEM III and IV grouts. In addition, the fall at later ages of capacitance C_1_, which was not observed for grouts hardened in distilled water, could indicate the appearance of microcracking, because the pore structure was no longer able to contain the expansive products formed during the sulphate attack.

The low frequency capacitance C_2_ has been associated with the pore surface in contact with electrolyte present in the material and it is related to the formation of CSH gel layers, which occupy the pores [65]. The hydration and pozzolanic reactions products are deposited on the pore surface and they increase the specific surface of the pores [45], because these products form rough structures. Moreover, it could be expected that those rough structures would progressively destroyed when the sulphate attack is advanced enough, which would imply a reduction of the capacitance C_2_.

For samples exposed to sodium sulphate solution (see Figure 6), the capacitance C_2_ showed a similar tendency than that observed for the rest of impedance spectroscopy parameters, previously described and discussed. The highest maximum values of this parameter have been noticed for grouts with slag and fly ash. This result would indicate that this samples would have a greater specific pore surface compared to CEM I ones, which would imply a more refined microstructure, corroborating the resistances R_1_ and R_2_ results. The decrease of capacitance C_2_ showed by the grouts since a certain age (see Figure 6) would mean a loss of specific pore surface and tortuosity of the porous network, which could be produced by the appearance of expansive products as a consequence of sulphate attack. These products would break the structures formed on the pore surface during the hydration and pozzolanic reactions.

In general, the capacitance C_2_ was higher for grouts hardened in distilled water than for those exposed to sodium sulphate solution (see Figure 7), although for each cement type in the short-term this parameter was very similar for both conditions. The optimum hardening condition, as it is the immersion in water distilled, facilitates the development of hydration and pozzolanic reactions, so the specific pore surface increases and the microstructure becomes more refined, as showed the greater values of capacitance C_2_ for grouts exposed to this condition. Besides, no loss of refinement is produced in the long-term, as indicated the fact that this capacitance kept practically constant. The similar behaviour of capacitance C_2_ in the short-term observed for grouts exposed to sodium sulphate solution could be due to a combined action of solid formation by the hydration and pozzolanic reactions and an initial silting of the pores produced by the formation of ettringite, which would increased the solid-electrolyte interphase and consequently rising the C_2_ values. In subsequent stages of the sulphate attack, the continuous ettringite formation would damage the rough structures formed on the pore surface, especially for smaller pores, causing a drop of capacitance C_2_.

The electrical resistivity results for samples exposed to sulphate solution were very similar than impedance resistance R_2_ ones (see Figure 8). Nevertheless, it is important to emphasize that the tendency change in the long-term evolution of resistivity is not as clear as has been observed for impedance resistance R_2_. This could be due to the different geometry of samples used for each technique, and in particular related to their different relationship between surface and volume. For impedance spectroscopy, 10 cm-diameter disks with 2 cm of thickness were exposed to sulphate medium, and for electrical resistivity, cylinders of 30 cm height and 7.5 cm diameter were tested. Therefore, the relationship surface/volume was approximately twice for impedance spectroscopy specimens than for resistivity ones. Then, if the relationship surface/volume is higher, it could be expected that the sulphate attack effects were visible at earlier ages, which would justify the fact that these effects have been detected before by impedance spectroscopy than by the monitoring of electrical resistivity, as has been observed in this research.

On the other hand, another explanation of this different behaviour of electrical resistivity, in which hardly decrease was observed at greater ages, could be the fact that this parameter has been measured using a superficial technique, as is Wenner four-point test. As a consequence, maybe it has been only registered the precipitation of sulphates in the most superficial pores of the samples, which would increase considerably the resistivity. This contrasted with impedance spectroscopy, which is a global technique and allows measurements through the specimens, not only on their surface, obtaining in this way more reliable information of pore network of the grouts. Finally, regarding the comparison between electrical resistivity results obtained for each storage condition studied (see Figure 8), the grouts immersed in distilled water showed a continuous rising tendency of this parameter, especially for CEM IV samples, while those exposed to sulphate solution tended to stabilize or even decrease in the long-term. The behaviour of grouts in distilled water medium would indicate the effects of progressive development of hydration and pozzolanic reactions, in accordance with previously discussed results. For grouts hardened in contact with sodium sulphate solution, the different behaviour of resistivity at greater ages would be related to the effects of sulphate attack in the microstructure, despite the already explained limitations of Wenner four-point test.

In relation to classical mercury intrusion porosimetry, the total porosity of grouts exposed to sodium solution decreased in the short-term (see Figure 9), which would indicate a solid formation and a reduction of total volume of pores. This result is in agreement with impedance spectroscopy and resistivity ones, and it could be a consequence of the new products formation through the development of slag and clinker hydration and fly ash pozzolanic reactions [5,13]. On the other hand, the small rise of total porosity observed for CEM III and IV grouts could be related to the sulphate attack effects, which would involve the formation of expansive products [19,20,22,72], producing a loss of solid fraction in the sample. These results were in line with those obtained for other authors [56,73]. Moreover, there was not a high difference between the total porosities obtained for the three types of grouts studied. This result is in keeping with the impedance capacitance C_1_ ones (see Figure 4), previously discussed, and therefore, they would indicate that the solid fraction was similar for all grouts, regardless of cement type used. The similar behaviour of total porosity and capacitance C_1_ would be justified because both parameters give information about the total proportion of solids in the material, without considering its pore size distribution [45].

The total porosity of grouts showed different results for each storage condition and cement type (see Figure 10). The total porosity of CEM I grouts was very similar for both media studied, and this could be related to the development of clinker hydration, especially for those exposed to an optimal condition, as immersion in distilled water. For CEM I grouts in contact with aggressive sulphate solution, the combined effect of clinker hydration and the pores silting caused by the precipitation of sulphates and other salts, as consequence of C_3_A reactions, could play an important role in their total porosity results. The beneficial effect of optimum hardening condition was more prominent for CEM III samples, and the immersion in distilled water would favour the development of slag hydration and new solids formation [45,74], as suggested the great fall of their total porosity with age, compared to those submerged in sulphate solution, in which the formation of expansive products would hinder a more considerable decrease of porosity. Finally, in relation to CEM IV grouts, the lowest total porosity corresponded to those hardened in contact with sulphate solution. This result could be due to the possible effect of Na_2_SO_4_ as an activator of pozzolanic reactions of fly ash, and it is agreement with other authors [75,76].

The pore size distributions of the grouts exposed to sulphate solution (see Figure 11) showed a progressive microstructure refinement with age, regardless of cement type used. These results are in keeping with those obtained in the short-time for impedance spectroscopy R_1_ and R_2_ resistances and electrical resistivity. They would point out the solid phases formation, mainly by the development of slag and clinker hydration and pozzolanic reactions of fly ash [5,11], so the pore size distribution is shifted towards finer pores. Furthermore, the microstructure was more refined for grouts with active additions than for CEM I ones, which is also in accordance with impedance spectroscopy and electrical resistivity results, and with other researches too [5,33,77], even for the exposure to sulphate attack [56]. At 28 days, the pore network of CEM III grouts was a little more refined than for CEM IV ones. This fact would be a result of the different behaviour of slag and fly ash. The hydraulic nature of slag allows that they start to react since the mixing of the grouts [5], while the pozzolanic nature of fly ash entails that its reactions start later than the beginning of clinker hydration [78], because they need the presence of portlandite formed in this hydration [10,11]. Then, a delay in microstructure refinement process is produced, as it was observed in the pore size distribution results, which is also agreement with the later increase of impedance parameters and electrical resistivity for CEM IV grouts, previously explained.

**Figure 15:**
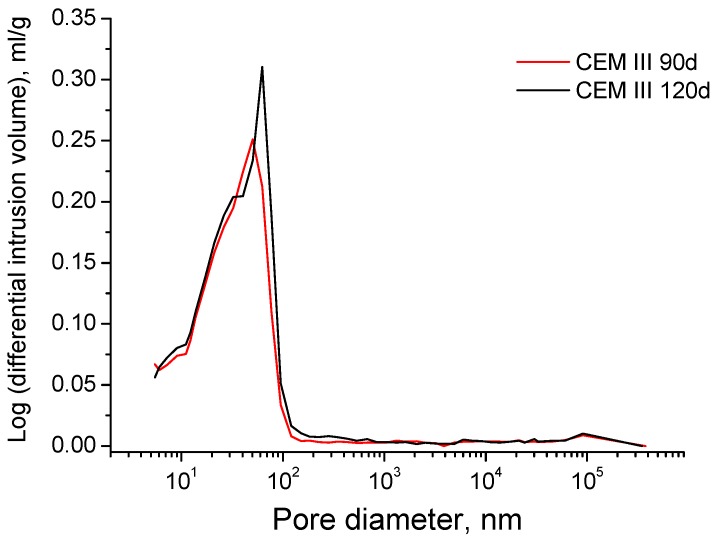
Curves of the logarithm of differential intrusion volume versus pore size obtained at 90 and 120 hardening days for CEM III grouts exposed to sodium sulphate solution.

**Figure 16:**
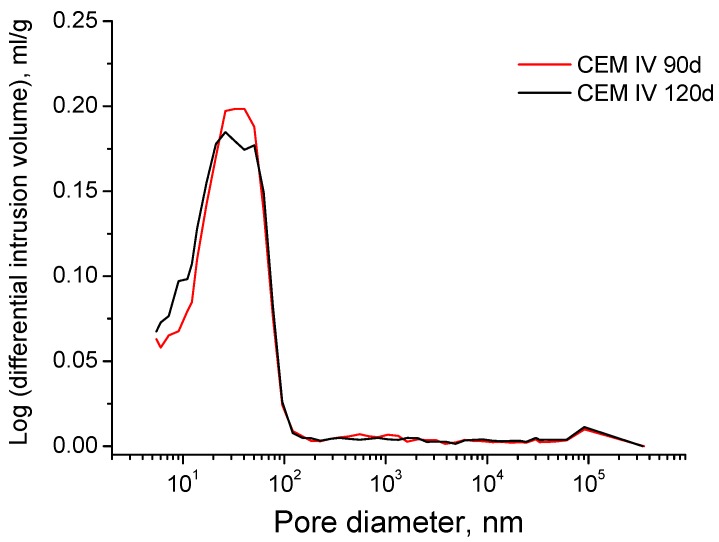
Curves of the logarithm of differential intrusion volume versus pore size obtained at 90 and 120 hardening days for CEM IV grouts immersed in sodium sulphate solution.

Nevertheless, no noticeable loss of pore network refinement has been noted in the long-term for grouts immersed in sulphate solution (see Figure 11), in contrast to impedance spectroscopy results. This result could be related to the drawbacks of mercury intrusion porosimetry [69,70] and it would suggest that more time would be needed for detecting the possible effects of sulphate attack in the pore size distribution of grouts using this technique. However, mercury intrusion porosimetry also allows studying the pore size distribution of the samples using the curves logarithm of differential intrusion volume versus pore size, in which the peaks reveal the presence of pores families. Then, in order to detect those possible effects of sulphate attack in pore size distribution of the grouts, these curves were also plotted (see Figure 15 and Figure 16).

For CEM III grouts, the main family of pores at 90 and 120 days, which is indicated by the highest peak of curves depicted in Figure 15, belonged to the pore range comprised between 10 and 100 nm. However, this family showed a higher pore size at 120 days, because the peak is closer to 100 nm at that age than at 90 days. Therefore, it is confirmed that a slight loss of pore network refinement was produced, which was not detected in the representation of pore size distribution using fixed pore ranges (see Figure 11). The curves logarithm of differential intrusion volume versus pore size for CEM IV grouts (see Figure 16) showed that the loss of microstructure refinement was not as clear as that noted for CEM III ones. As can be observed in Figure 16, for CEM IV samples only one family of pores existed in the range from 10 to 100 nm at 90 days, which was wider than that observed for CEM III ones. This only family developed in two different families at 120 days, as indicated the fact that the peak observed at 90 days was divided in two ones at 120 days. This could be due to the combined effect of the sulphate attack, which would start to reduce the microstructure refinement, and the new solids formation as a products of fly ash pozzolanic reactions, which would be still developing because they are delayed in comparison to clinker hydration [10,11]. On the one hand, the effects of expansive products produced by sulphate attack would move the original 90 days peak towards higher pore sizes, giving room to the right peak observed at 120 days in Figure 16. On the other hand, the products of the delayed pozzolanic reactions of fly ash would tend to move the 90 days peak towards smaller pore sizes, leading to the left peak at 120 days in Figure 16. This could explain the evolution of the curve logarithm of differential intrusion volume versus pore size observed for CEM IV grouts, and the appearance of two peaks observed at 120 days.

Regarding the comparison between the pore size distributions of the grouts exposed to both media studied (see Figure 12), for CEM I and III grouts the microstructure refinement was higher for those hardened in contact with distilled water. This result could be related to the formation of expansive products during the sulphate attack, which would produce cracks in the pore network, reducing the percentage of smaller pores. On the other hand, for CEM IV grouts, the microstructure was more refined for those immersed in sodium sulphate solution than for those stored in distilled water. This result is in line with total porosity ones, and it could be due to the effect of pozzolanic activation of fly ash produced by the sodium sulphate [75,76].

The Hg retained in the sample at the end of the MIP test gives information about the tortuosity of the pore network [60]. In general, the results of this parameter obtained for the grouts exposed to sulphate attack (see Figure 13) are in keeping with the rest of MIP results previously discussed. The highest values of Hg retained observed for grouts with active additions would mean a greater tortuosity of the microstructure, which coincides with their higher pore refinement and with their greater specific pores surface, suggested by impedance spectroscopy capacitance C_2_ results. The fall at later ages of Hg retained (observed first for CEM III grouts and later for CEM IV ones) is also in accordance with the majority of results of this research, and it could be associated to the damage produced by sulphate expansive products in the pore structure of the samples, which would reduce its tortuosity. Moreover, it is important to emphasize that for CEM III grouts practically coincided the age (between 50 and 60 days approximately) when Hg retained and impedance spectroscopy capacitance C_2_ started to fall. Lastly, comparing the Hg retained results for both storage conditions studied, it is interesting to note that the highest values of this parameter for CEM IV samples were obtained for those immersed in sulphate solution. This result would confirm again the important effect of Na_2_SO_4_ as an activator of pozzolanic reactions of fly ash [75,76].

Overall, the results of microstructure characterization carried out using impedance spectroscopy, mercury intrusion porosimetry and electrical resistivity are in agreement. They showed a first stage of microstructure refinement as a consequence of the development of clinker and slag hydration and fly ash pozzolanic reactions. Furthermore, the ettringite formation due to sulphate attack could initially contribute to this pore network refinement, until the pores were filled enough. After that, it would start a second stage, in which the damages in the microstructure produced by expansive products become more important, and it could be supposed that they would be the major process which would control the evolution of the microstructure, although the hydration and pozzolanic reactions could be still producing. During this second phase, a loss of microstructure refinement is produced, showed mainly by the decrease of impedance spectroscopy parameters. The abovementioned mechanism is in keeping with that proposed by other authors [79].

Finally, the compressive strength results at 28 days (see Table 3) are influenced by the different strength type of cement used, although the grouts which incorporate active additions showed a smaller loss of strength in sodium sulphate media than CEM I ones, compared to distilled water medium. Then, the highest values of this parameter have been observed for CEM I grouts as it was expected, because it was used a high early strength cement type. Although the strength type of slag cement was the same than for CEM I, the compressive strength of CEM III grouts was lower, which was also expected because the slag cement was low early strength type. At last, the smallest compressive strength was observed for CEM IV grouts, due to the lowest strength type of this cement.

## 5. Conclusions

The main conclusions that can be drawn from the results previously discussed can be summarized as follows:

- In general, slag and fly ash cement grouts for micropiles showed a more refined microstructure than CEM I ones for both sodium sulphate solution and distilled water hardening media.
- In the short-term (until approximately 40 hardening days), the microstructure of the grouts exposed to sodium sulphate solution becomes more refined, due to the formation of solid phases as products of clinker and slag hydration and fly ash pozzolanic reactions. Moreover, the ettringite formation as a consequence of sulphate attack would also contribute to this microstructure refinement.
- In the long-term (approximately from 60–90 days, depending on the cement type, up to 120 days of exposure), for grouts in contact with sodium sulphate solution, when enough silting of pores by expansive products formation has been produced, the continuous sulphate attack would damage the rough structures formed in the pore surface, causing a loss of microstructure refinement, independently that the hydration and pozzolanic reactions could be still producing.
- The exposure of grouts to an optimum hardening condition, like the immersion in distilled water, favours the development of hydration and pozzolanic reactions and improves the development of their microstructure, reducing the porosity and increasing the refinement of the pore network.
- The development of microstructure for CEM IV grouts was delayed in comparison with CEM I and III ones. This could be associated with the later beginning of fly ash pozzolanic reactions with respect to clinker and slag hydration.
- The compressive strength of the grouts at 28 days was influenced by the different strength type of cement used. However, the loss of compressive strength for samples submerged in sodium sulphate solution, compared to those hardened in distilled water, was lower for slag and fly ash cement grouts than for CEM I ones.
- Fly ash grouts exposed to sodium sulphate solution showed a better behaviour in relation to microstructure development, compared to those submerged in distilled water. This could be due to the possible effect of 15% Na_2_SO_4_ as an activator of pozzolanic reactions of fly ash.
- In general, the microstructure characterization carried out using impedance spectroscopy, mercury intrusion porosimetry and electrical resistivity are in agreement. However, it seems that non-destructive impedance spectroscopy is the most sensitive technique for following the changes in the porous network of slag and fly ash cement grouts for micropiles exposed to an aggressive sodium sulphate medium.
- In view of the results obtained in this research, the micropiles made using fly ash and slag cement grouts could have a good performance in contact with aggressive sodium sulphate media, even better than ordinary Portland cement ones.

## Figures and Tables

**Table 1:** Mix proportions of the grouts per cubic meter.

Element	Kg/m^3^
Cement	1270
Distilled water	635

**Table 2:** Samples used for each technique and exposure media.

Technique	Exposure Medium	Type of Sample	Exposure Time	Samples Tested	Standard
Impedance spectroscopy	15 wt % Na_2_SO_4_	Disks 10 cm diameter and 2 cm thickness	Up to 120 days	5	n/a
	Distilled water	Disks 10 cm diameter and 1 cm thickness	Up to 90 days	5	n/a
Electrical resistivity	15 wt % Na_2_SO_4_	Cylinders 30 cm height and 7.5 cm diameter	Up to 120 days	4	UNE 83988-2
	Distilled water	Cylinders 30 cm height and 15 cm diameter	Up to 90 days	4	UNE 83988-2
Mercury intrusion porosimetry	15 wt % Na_2_SO_4_	Pieces of disks 10 cm diameter and 2 cm thickness	28, 60, 90 and 120 days	2	n/a
	Distilled water	Pieces of disks 10 cm diameter and 1 cm thickness	2, 28 and 90 days	2	n/a
Compressive strength	15 wt % Na_2_SO_4_	Prisms 4 cm × 4 cm × 16 cm	28 days	3	UNE-EN 196-1
	Distilled water	Prisms 4 cm × 4 cm × 16 cm	28 days	3	UNE-EN 196-1

**Table 3:** The 28-day compressive strength for studied cement grouts (standard deviation in brackets).

Cement Type	Exposure Medium	
	Distilled Water	15% Na_2_SO_4_
CEM I	49.7 MPa (1.56)	43 MPa (2.65)
CEM III	42.7 MPa (2.23)	38.7 MPa (2.08)
CEM IV	31.1 MPa (1.88)	30.1 MPa (1.39)
